# Apatite Formation and Biocompatibility of a Low Young’s Modulus Ti-Nb-Sn Alloy Treated with Anodic Oxidation and Hot Water

**DOI:** 10.1371/journal.pone.0150081

**Published:** 2016-02-25

**Authors:** Hidetatsu Tanaka, Yu Mori, Atsushi Noro, Atsushi Kogure, Masayuki Kamimura, Norikazu Yamada, Shuji Hanada, Naoya Masahashi, Eiji Itoi

**Affiliations:** 1 Department of Orthopaedic Surgery, Tohoku University Graduate School of Medicine, Sendai, Miyagi, Japan; 2 Institute for material research, Tohoku University, Sendai, Miyagi, Japan; Institute for Frontier Medical Sciences, Kyoto University, JAPAN

## Abstract

Ti-6Al-4V alloy is widely prevalent as a material for orthopaedic implants because of its good corrosion resistance and biocompatibility. However, the discrepancy in Young’s modulus between metal prosthesis and human cortical bone sometimes induces clinical problems, thigh pain and bone atrophy due to stress shielding. We designed a Ti-Nb-Sn alloy with a low Young’s modulus to address problems of stress disproportion. In this study, we assessed effects of anodic oxidation with or without hot water treatment on the bone-bonding characteristics of a Ti-Nb-Sn alloy. We examined surface analyses and apatite formation by SEM micrographs, XPS and XRD analyses. We also evaluated biocompatibility in experimental animal models by measuring failure loads with a pull-out test and by quantitative histomorphometric analyses. By SEM, abundant apatite formation was observed on the surface of Ti-Nb-Sn alloy discs treated with anodic oxidation and hot water after incubation in Hank’s solution. A strong peak of apatite formation was detected on the surface using XRD analyses. XPS analysis revealed an increase of the H_2_O fraction in O 1s XPS. Results of the pull-out test showed that the failure loads of Ti-Nb-Sn alloy rods treated with anodic oxidation and hot water was greater than those of untreated rods. Quantitative histomorphometric analyses indicated that anodic oxidation and hot water treatment induced higher new bone formation around the rods. Our findings indicate that Ti-Nb-Sn alloy treated with anodic oxidation and hot water showed greater capacity for apatite formation, stronger bone bonding and higher biocompatibility for osteosynthesis. Ti-Nb-Sn alloy treated with anodic oxidation and hot water treatment is a promising material for orthopaedic implants enabling higher osteosynthesis and lower stress disproportion.

## Introduction

Ti-6Al-4V alloy is widely used for orthopaedic implants because of its corrosion resistance and biocompatibility [1]. However, its Young’s modulus is 110 GPa, considerably greater than that of human cortical bone (10–30 GPa) [2]. In clinical uses, the difference between the Young’s modulus of a total hip arthroplasty prosthesis and that of cortical bone can induce stress disproportion and cause thigh pain [3] [4]. To resolve this problem, β-type titanium alloys with a low Young’s modulus have been developed as biomedical materials [5] [6] [7] [8] [9] [10] [11] [12] [13] [14] [15] [16] [17] [18] [19] [20]. A new β-type Ti-Nb-Sn alloy with a lower Young’s modulus (less than 50 GPa), considerably close to that of human cortical bone, has the potential to reduce likelihood of stress shielding and thigh pain [12]. Moreover, Ti-Nb-Sn alloys have the unique property that their stiffness and Young’s moduli are gradually increased by heating. The Young’s moduli of Ti-Nb-Sn alloys are, therefore, adjustable and they are expected to have high bonding strength with bone as biomedical materials [9] [11] [12]. Heating at temperatures above 673 K induces an increased Young’s modulus in Ti-Nb-Sn alloys [21]. We previously reported that the Ti-Nb-Sn alloy had greater biocompatibility, as compared with that of the Ti-6Al-4V alloy [22]. The Ti-Nb-Sn alloy showed better bone induction ability in an experimental model [22].

Certain surface modifications such as plasma-sprayed coating, hydrogen peroxide hydrothermal treatment and alkali-heat treatment have been applied to induce rapid and secure bone integration [23] [24] [25] [26] [27]. Among them, anodic oxidation (AO) is expected to enable apatite formation on the surface of titanium alloys. Anodic oxide on pure titanium (CP-Ti), prepared in a sulfuric acid electrolyte followed by annealing at 450°C for 5 h in air, led to formation of a good apatite layer on the CP-Ti in simulated body fluid (SBF) [28]. The surface of the Ti substrate was covered by a porous oxide film, comprising titania with crystalline structures of anatase and rutile. The investigators reporting this proposed that a certain amount of titania with anatase and/or rutile on the oxidized titanium surface was essential for apatite formation. Furthermore, it was shown that hot water (HW) treatment of CP-Ti after AO in an acetic acid electrolyte promoted apatite formation in SBF [29]. In that study, the surface was characterized by anatase-structured TiO_2,_ exhibiting hydroxyl group adsorption, and this was required for apatite nucleation and deposition in SBF. For Ti-Nb-Sn alloys, HW treatment after AO has the potential to induce apatite formation on the surface in SBF without affecting the low Young’s modulus.

The purpose of this study was to investigate apatite-forming and bone-bonding abilities *in vivo* of a Ti-Nb-Sn alloy treated with AO in acetic acid solution followed by HW. We hypothesized that this combination treatment would improve both apatite formation and bone-bonding abilities, as compared with the properties of untreated Ti-Nb-Sn alloy.

## Materials and Methods

### Preparation of Ti–Nb–Sn alloy disc and rod

Ti-25Nb-11Sn (wt.%) alloy ingots were prepared by a high frequency induction in an Ar atmosphere using 99.9% titanium, 99.9% niobium and 99.9% tin. The melted ingot was cut into several pieces and vacuum arc re-melted to homogenize its chemical composition, followed by hot forging at 1100°C, cold rolling and swaging to a rod with a 15-mm diameter. This specific alloy with a Young’s modulus lower than 55 GPa is referred to as “Ti-Nb-Sn alloy” hereafter.

For the evaluation of apatite formation in SBF, Ti–Nb–Sn alloy disc samples of 10 mm diameter and 2.0 mm thickness were prepared from the 15 mm rod. As a control, disc samples were prepared from CP-Ti (grade 2). Each disc was polished using SiC grinding paper of 800 grit then rinsed in ethanol in an ultrasonic cleaner and dried in air.

The cylindrical rods of 4.5 mm in diameter and 32 mm in length were prepared from the 8 mm diameter rod by turning on a lathe for the pull-out test. The machined surface had a roughness of Ra 6.04 μm. The dynamic Young’s modulus of the Ti–Nb–Sn alloy was 45.6 GPa. The rod had a protruding portion (4.0 mm diameter, 6 mm length) with a transverse hole for mechanical testing and a capped tapering portion on the other end (3-mm end diameter, 1-mm length) so as to be smoothly inserted into the medullary canal. The rods were produced with a machined surface finish and stabilized with nitric acid. All implants were sterilized in an autoclave.

### Surface modification

Both Ti-Nb-Sn alloy and CP-Ti discs were anodized for 0.5 h at room temperature in a 2.0 M acetic acid solution potentiostatically controlled at a voltage of 200 V and current of 50 mA/cm^2^. These were designated as AO-treated discs. After AO treatment, the discs were immersed in 15 ml of distilled water at 80°C for 48 h (HW treatment) and subsequently dried in a dry incubator at 36.5°C for 24 h. These treated discs were designated as AO- plus HW-treated discs.

Ti-Nb-Sn alloy rods were also anodized in an acetic acid electrolyte. In this study, AO was performed for 0.5 h at room temperature in 2.0 M acetic acid solution potentiostatically controlled at a voltage of 500 V with a current of 50 mA/cm^2^. Anodized rods were immersed in 15 ml distilled water at 80°C for 48 h and these are designated as AO- plus HW-treated rods.

All Ti-Nb-Sn alloy discs and rods treated with AO and HW were incubated in 25 ml Hank’s solution (Hank’s Balanced Salt Solution, GIBCO, Grand Island, NY, USA) at 36.5°C for 7 days as previously reported. Ionic concentrations in Hank’s solution are almost equivalent to those in human blood plasma [30]. After incubation for 7 days, these discs and rods were removed from Hank’s solution, gently washed with distilled water and dried in an incubator for 24 h.

### Surface analyses

Ti-Nb-Sn alloy and CP-Ti discs were analysed by field-emission scanning electron microscopy (FE-SEM, Scanning Electron Microscope SU 8000, Hitachi, Japan) and X-ray diffraction (XRD, X’Pert diffractometer, PANalytical, Netherlands) with a thin-film geometry arrangement at 0.5° glancing angle and a rotating detector. X-ray photoelectron spectroscopy (XPS) measurements were conducted with an electron spectrometer (Kratos AXIS-Ultra DLD, Shimadzu, Japan) equipped with monochromated Al Kα radiation at a base pressure of 3.0×10^−7^ Pa. The full width at half maximum intensity of the Ag 3d_5/2_ peak is 0.73 eV. Measurements were performed with both AO-treated and AO-plus HW-treated discs, as indicated in the figures (six specimens for each groups). The O 1s spectra were deconvoluted into three individual spectra [31]; metal oxide at a binding energy of about 530 eV, hydroxyl groups at about 532 eV and H_2_O at about 533.5 eV, respectively. After incubation in Hank’s solution for 7 days, the discs were analysed using FE-SEM and XRD to investigate apatite nucleation. Rods soaked in Hank’s solution were assessed for formation of apatite with FE-SEM.

### Animal experiments

Adult male Japanese white rabbits were purchased from Charles River Japan. All rabbits were housed in the Animal Unit of Tohoku University Medical School, an environmentally controlled and specific pathogen-free facility. Animal protocols were reviewed and approved by the Tohoku University Animal Studies Committee. All experiments were performed using rabbits weighing 3.0–3.5 kg. AO-plus HW-treated and untreated Ti-Nb-Sn alloy rods were inserted into the medullary canal of rabbit femurs as previously described [22]. The animals were maintained in individual cages (60 × 51 × 35 cm) (W × D × H) at 22 ± 2°C and 40 ± 20% humidity, receiving water and specific animal pellet-type laboratory-animal food. After premedication with ketamine (25 mg/kg) by intramuscular injection, the animals were anaesthetized with ketamine (10 mg/kg) and xylazine (3 mg/kg) by intravenous injection. Cefazolin (30 mg/kg) was administered by intravenous injection before surgery. The postoperative wound conditions (visually healing, oedema of soft tissues) were estimated. The animals were sacrificed using intravenous pentobarbital (120 mg/kg) 3 and 6 weeks after rod implantation.

### Pull-out test for bone-bonding strength

The distal end of the femoral condyle was excised, exposing the protruding portion for attachment to the testing machine (Autograph, Shimadzu, Japan). A universal connecting device was constructed to allow the testing machine to pull the rod vertically. The failure load was measured for each of AO-plus HW-treated rods and untreated rods after keeping them in rabbits for 3 and 6 weeks [22].

### Histomorphometric analysis

**Fig 1:**
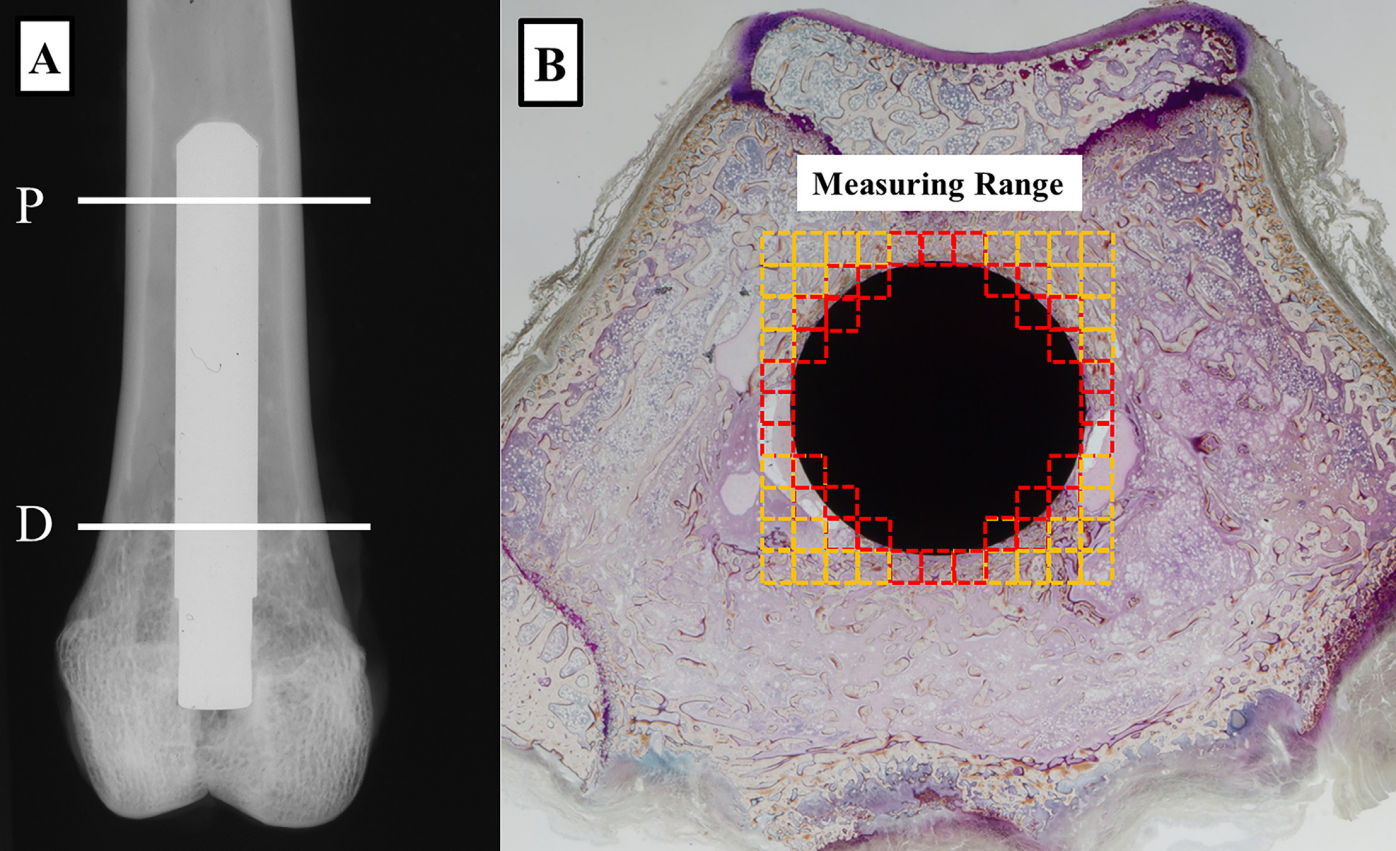
Regions of histomorphometric measurements. (A) Radiograph of a Ti-Nb-Sn rod implanted in the distal femur of a rabbit. Locations of proximal (P) and distal (D) samples subjected to histological analyses are indicated. (B) A histological image of a Ti-Nb-Sn rod-implanted femur. Red squares indicated regions of interest for quantitative histological analyses.

Histomorphometric analysis was performed 6 weeks after rods had been implanted in rabbits (n = 3 for each groups). Rabbit femurs were labelled with tetracycline (Sigma, Germany) and calcein (Dojindo Laboratories, Japan). Tetracycline (20 mg/kg) was subcutaneously injected for double-labelling at 7 and 2 days before implantation, and calcein (10 mg/kg) was subcutaneously injected at 7 and 2 days before sacrifice. Femurs with implants were excised and immediately placed in 70% ethanol and fixed for 5 days. Samples were stained for 6 days with Villanueva bone stain, dehydrated in ascending grades of ethanol, defatted in an acetone/methyl methacrylate monomer mixture (1:3) and embedded in methyl methacrylate (Wako Chemicals, Japan) without decalcification. Cross-sections (200 μm) were cut with a precision bone saw perpendicular to the long axis of the implant at two levels, proximal and distal, of the femur (Fig 1A). Sections were mounted on plastic slides and ground using a precision lapping machine (Maruto, Japan). Finally, specimens were manually ground to a thickness of 40 μm with monitoring under the microscope using the method described by Frost [32].

Histomorphometric measurements were made on each group of rods using a semiautomatic image analysing system (System Supply, Ina, Japan). The following indices for cancellous bone around a metal were estimated (Fig 1B): bone volume/tissue volume (BV/TV, %); trabecular thickness (Tb.Th, mm); osteoid volume/TV (OV/TV, %); osteoclast surface/bone surface (Oc.S / BS, %); osteoblast surface/BS (Ob.S / BS, %); mineral apposition rate (MAR, mm/day) [33].

### Statistical analysis

Statistical analysis was performed using JMP, Version 10 (SAS, Cary, NC, USA). All data are expressed as means ± SD. Statistically significant differences between values were determined using the Student’s t-test. Differences were considered significant at p<0.05.

## Results

### Surface analyses

**Fig 2:**
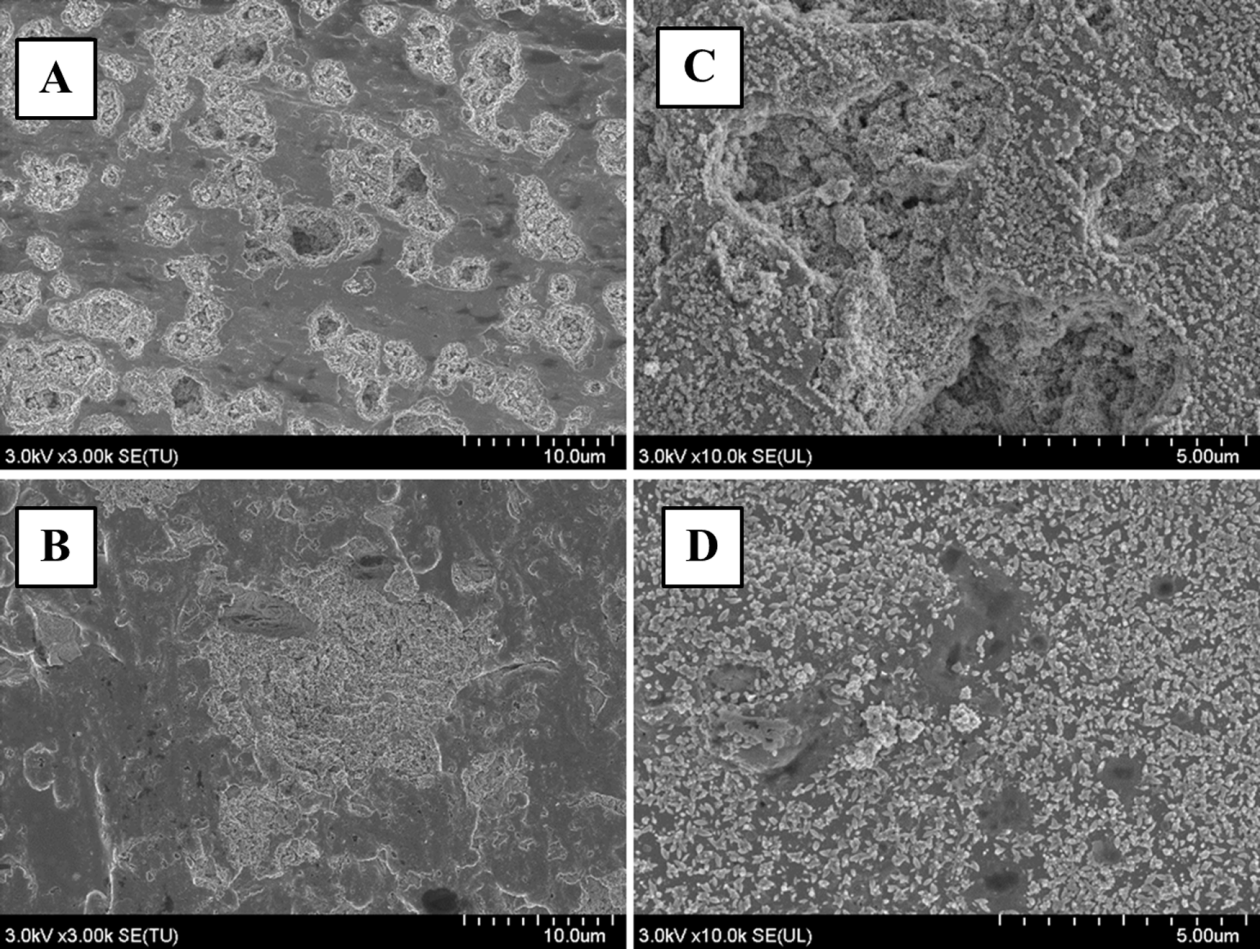
SEM images of AO- and HW-treated Ti-Nb-Sn and CP-Ti discs. Representative images of SEM micrographs of AO- and HW-treated Ti-Nb-Sn and CP-Ti discs. Numerous small spheres were observed on both substrates after HW treatment. (A) AO-treated CP-Ti; (B) AO-treated Ti-Nb-Sn; (C) AO- plus HW-treated CP-Ti; (D) AO- plus HW-treated Ti-Nb-Sn

The anodized oxide on both Ti-Nb-Sn and CP-Ti exhibit porous microstructure. The pore diameters of the oxide on the Ti-Nb-Sn (10 μm) were greater than those on the CP-Ti discs (1–3 μm) (Fig 2A and 2B). HW treatment after AO produced numerous small spheres on the surface of both CP-Ti and Ti-Nb-Sn alloy (Fig 2C and 2D).

**Fig 3:**
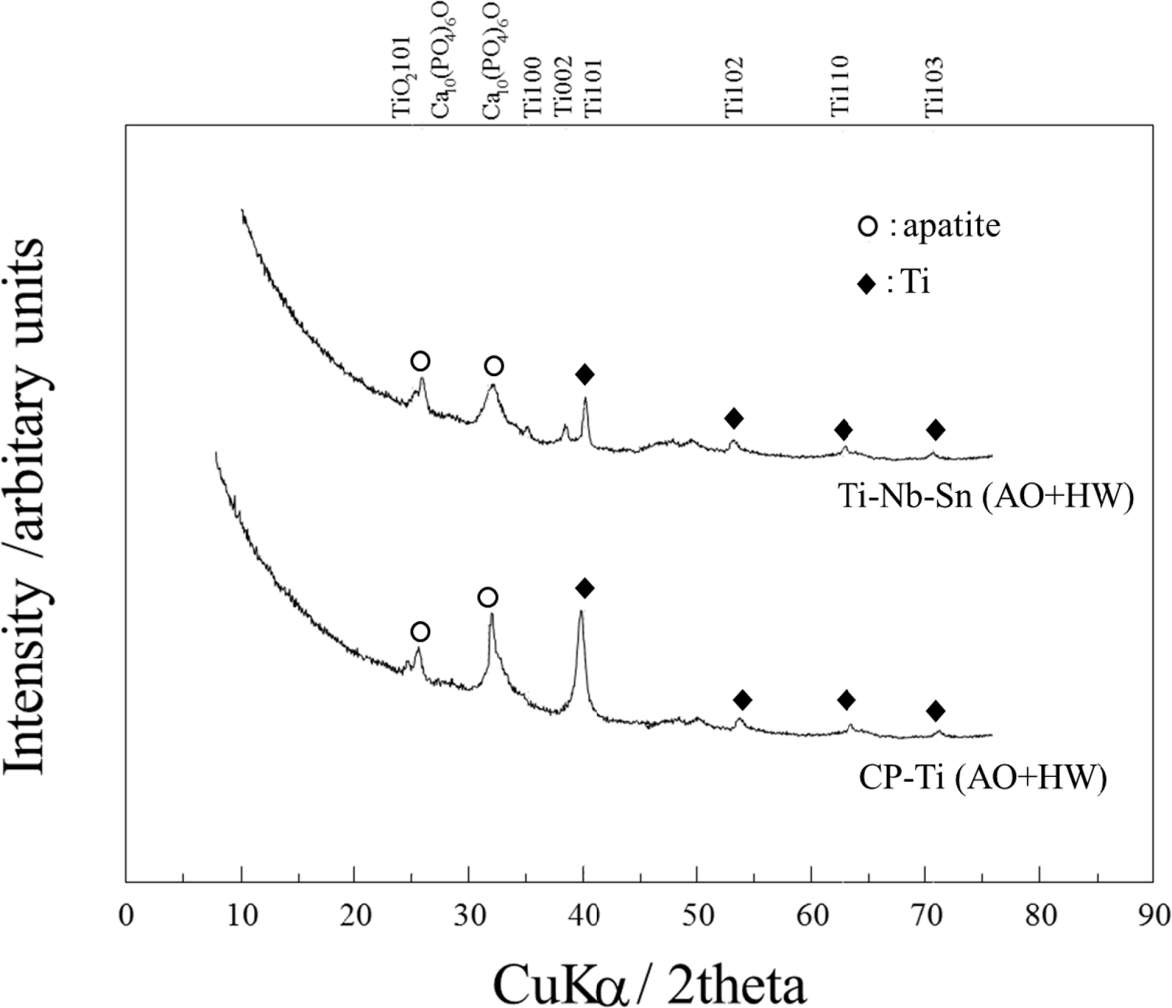
XRD analysis for apatite formation. XRD profiles of AO-treated and AO- plus HW-treated Ti-Nb-Sn and CP-Ti discs. After 7 days incubation in Hank’s solution, both CP-Ti and Ti-Nb-Sn alloy discs showed crystalline apatite formation.

Fig 3 shows XRD patterns on the surface of both CP-Ti and Ti-Nb-Sn alloy after AO-treatment and subsequent HW-treatment. After 7 days’ incubation in Hank’s solution, a crystalline apatite phase was observed on the surface of both CP-Ti and Ti-Nb-Sn alloy discs treated by AO plus HW.

**Fig 4:**
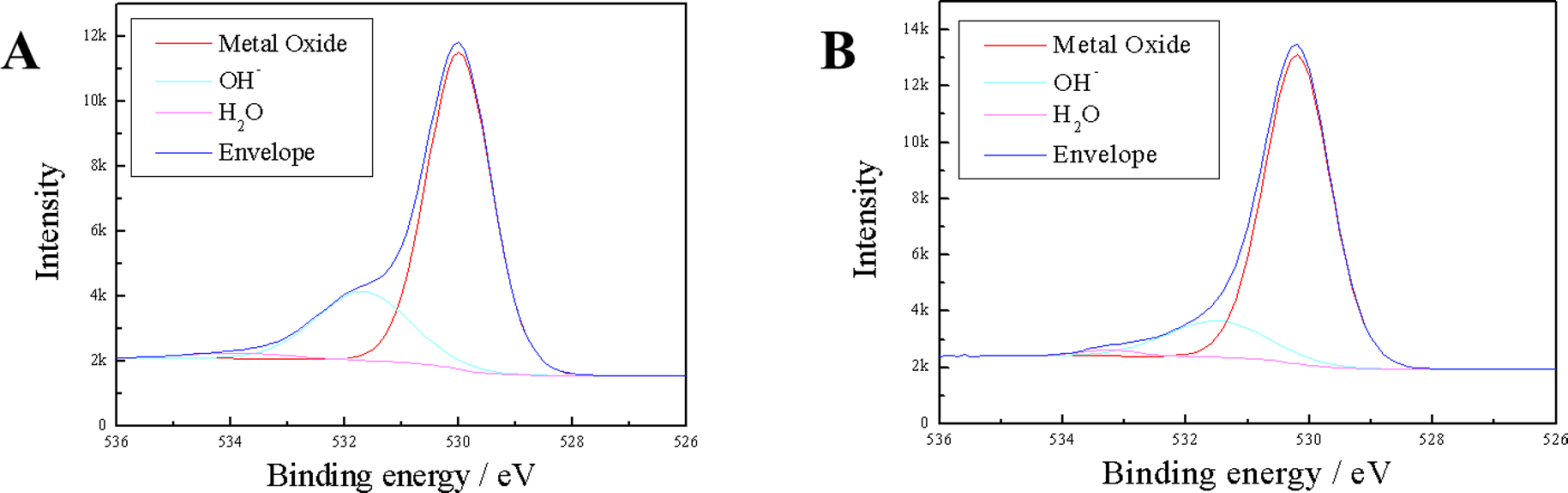
XPS analysis of the surface of Ti-Nb-Sn. O 1s XPS profiles of AO-treated and AO- plus HW-treated Ti-Nb-Sn. (A) AO-treated Ti-Nb-Sn; (B) AO- plus HW-treated Ti-Nb-Sn.

The survey XPS spectra of the anodic oxides on Ti-Nb-Sn showed Ti, Nb, Sn, O and N peaks. N was detected in the corresponding XPS spectra of the anodic oxide on CP-Ti and originated from contamination and air exposure during sample preparation. Narrow spectral analysis revealed that the oxide on Ti-Nb-Sn was composed of TiO_2_, Nb_2_O_5_ and SnO or SnO_2_ as described in reference [34]. The O 1s XPS was asymmetrical with a shoulder peak extending towards higher binding energies, which was due to hydroxyl groups (Fig 4A and 4B).

Table 1 summarizes the chemical composition calculated from XPS spectra. HW treatment did not change the fraction of hydroxyl groups of the anodic oxide on both CP-Ti and Ti-Nb-Sn significantly. Furthermore, we found that HW treatment significantly increased the fraction of H_2_O of the anodic oxide regardless of the substrate.

**Table 1:** Analyses of chemical composition of the surface layer calculated from the XPS spectra.

	Cp-Ti-AO	Cp-Ti-HW	p	TNS-AO	TNS-HW	p
O 1s Metal Ox	52.0 (0.56)	49.6 (3.08)		54.7 (1.99)	57.1 (1.52)	
O 1s OH-	16.1 (0.66)	17.0 (3.12)	0.59	14.0 (2.43)	11.5 (2.16)	0.07
O 1s H_2_O	1.4 (0.22)	2.5 (0.69)	0.015*	0.4 (0.29)	1.0 (0.25)	0.019*
Ti 2p	30.0 (0.1)	29.0 (0.9)		27.7 (0.95)	21.6 (0.47)	
N 1s	0.4 (0.04)	1.9 (1.42)		0.3 (0.27)	0.5 (0.25)	
Nb 3d				2.5 (0.39)	7.5 (0.1)	
Sn 3d				0.3 (0.09)	0.9 (0.07)	

The calculated chemical composition derived from XPS spectra for the surfaces of the anodic oxides before and after HW treatment. The data are averages of the measurements of six samples taken for each group, with standard deviations given in parentheses.

(*: p < 0.05).

**Fig 5:**
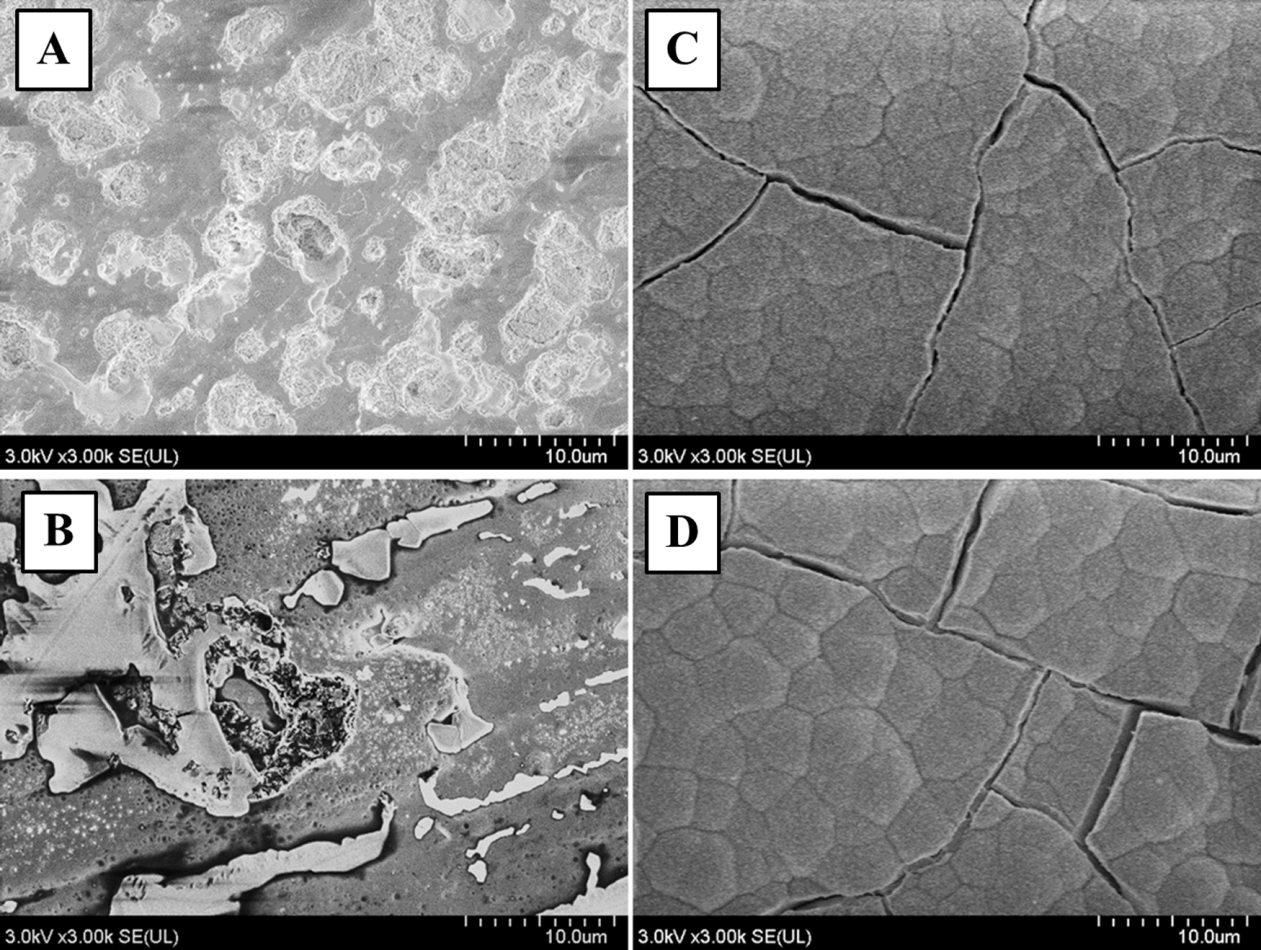
SEM images of apatite formation. Representative images of SEM micrographs of Ti-Nb-Sn and CP-Ti discs after AO-treatment or AO- plus HW-treatment, followed by 7 days’ incubation in Hank’s solution. A crystalline apatite layer is observed on the surface of Ti-Nb-Sn and CP-Ti discs after AO- plus HW-treatment and incubation in Hank’s solution. (A) AO-treated CP-Ti; (B) AO-treated Ti-Nb-Sn; (C) AO- plus HW-treated CP-Ti; (D) AO- plus HW-treated Ti-Nb-Sn. (A–D) were all incubated in Hank’s solution.

After 7 days’ incubation in Hank’s solution, the AO-treated groups showed no apatite formation on their surfaces in SEM images (Fig 5A and 5B). In contrast, numerous apatite nucleations and depositions were visible on the surfaces in the groups of Ti-Nb-Sn alloy and Cp-Ti that were AO- plus HW-treated with 7 days incubation in Hank’s solution (Fig 5C and 5D).

### Results from SEM images of Ti-Nb-Sn rods with apatite formation and pull-out test

**Fig 6:**
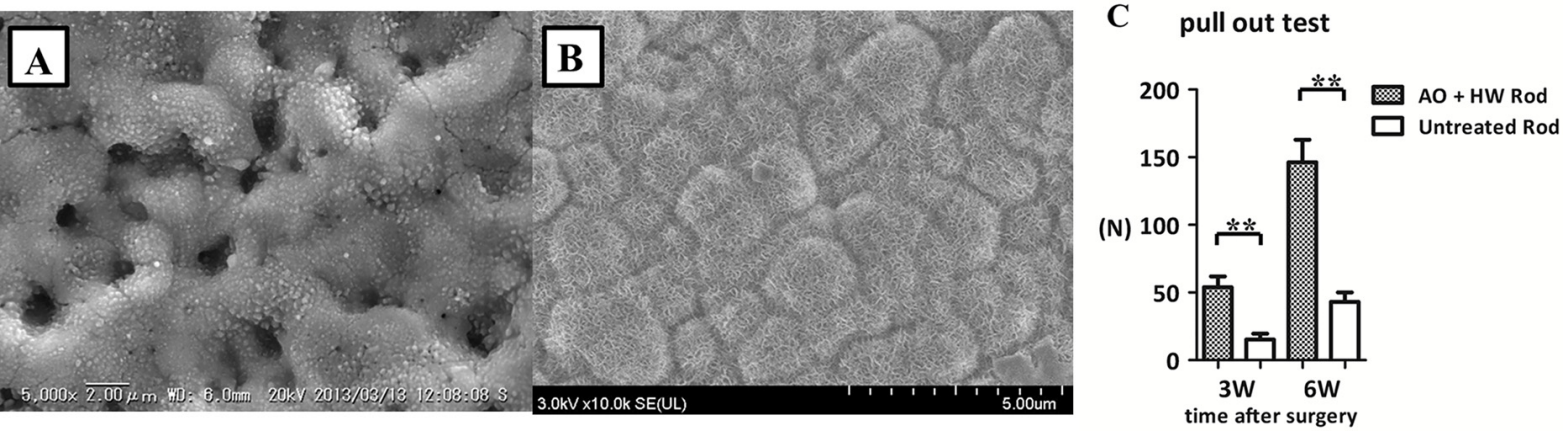
SEM images of Ti-Nb-Sn rods with apatite formation and pull-out test. Representative images of SEM micrographs of AO-treated and AO- plus HW-treated rods and the results of pull-out test of untreated and AO- plus HW-treated rods. (A) SEM images of AO-treated rod with incubation in Hank’s solution. Numerous small spheres were observed on the surface. (B) SEM images of AO- plus HW-treated rod with incubation in Hank’s solution. A crystalline apatite phase was observed. (C) Failure loads at 3 and 6 weeks after rod implantation were measured by the pull-out test. The failure loads of AO- plus HW-treated rods were significantly higher than those of untreated rods at 3 and 6 weeks. (**: p < 0.01).

Fig 6A and 6B show representative SEM images of the AO- treated and AO-plus HW-treated rods after 7 days’ incubation in Hank’s solution. SEM images of the AO- plus HW-treated rods with Hank’s solution incubation showed abundant apatite formation. SEM images of AO-treated rods with incubation in Hank’s solution did not indicate apatite formation. At 3 weeks after implantation, the failure load of AO- plus HW-treated rods, 54.0 ± 19.5 N, was significantly higher than that of untreated rods, 15.2 ± 10.7 N (p < 0.01). At 6 weeks after implantation, there was a significant difference (p < 0.01) between the failure load of AO- plus HW-treated rods, 146.3 ± 40.6 N, and that of untreated rods, 40.3 ± 17.2 N (Fig 6C). These results suggested that AO- plus HW-treatment induced apatite formation and improved bone-bonding ability and biocompatibility of Ti-Nb-Sn alloy rods.

### Histological evaluation

**Fig 7:**
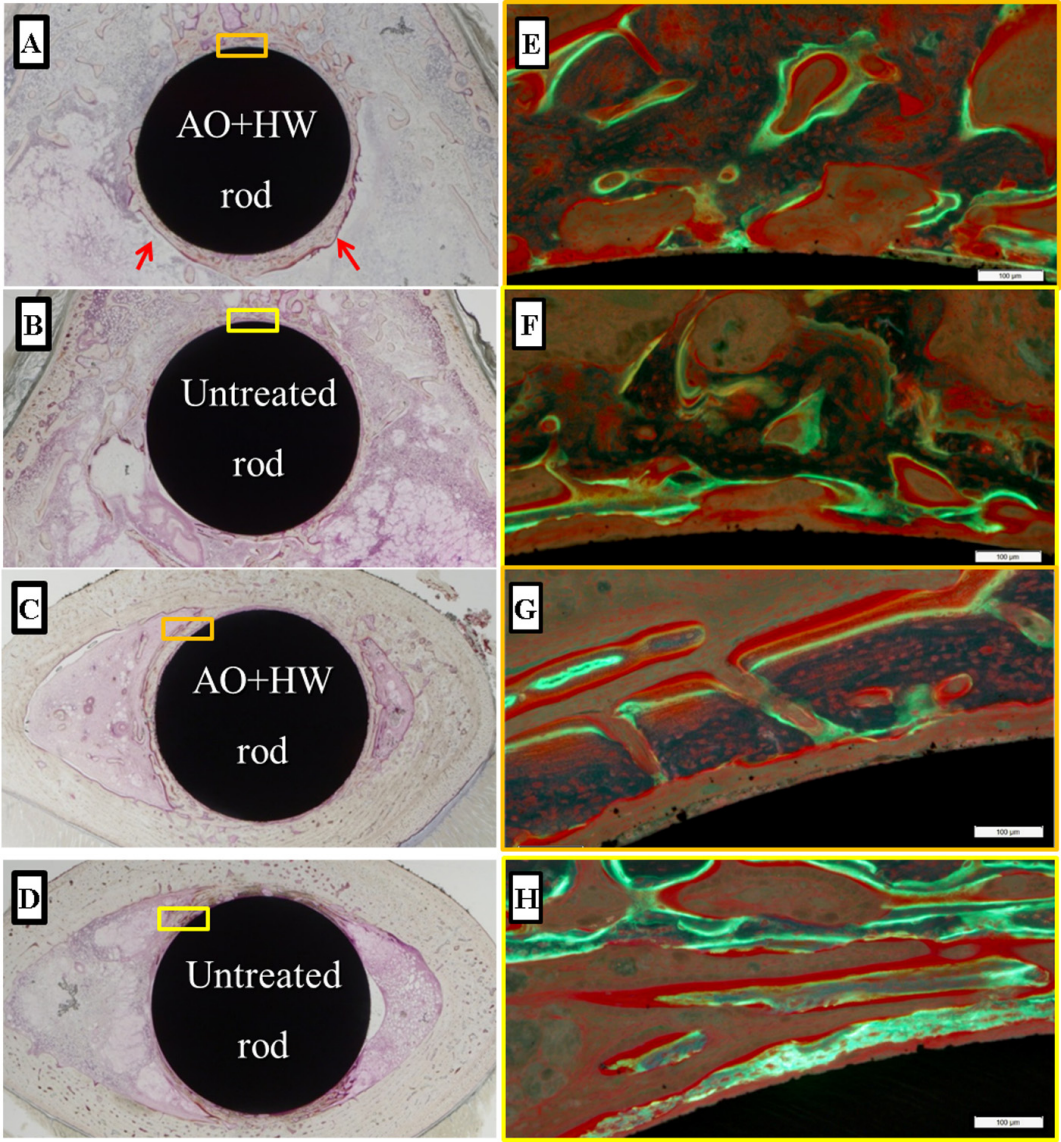
Histological images of newly-formed bone. Representative histological images of Ti-Nb-Sn alloys implanted into the femur. Newly formed bone was observed, especially in the area of the distal femur (arrows). Higher magnification images (panels E–H) of the rectangular areas (panels A–D) as visualized under a fluorescence microscope. The yellow and green fluorescence indicates tetracycline (injected before operation) and calcein (injected before death) signals, respectively. (A, E) Distal section of an AO- plus HW-treated rod; (B, F) Distal section of an untreated rod; (C, G) Proximal section of an AO- plus HW-treated rod; (D, H) Proximal section of an untreated rod.

**Fig 8:**
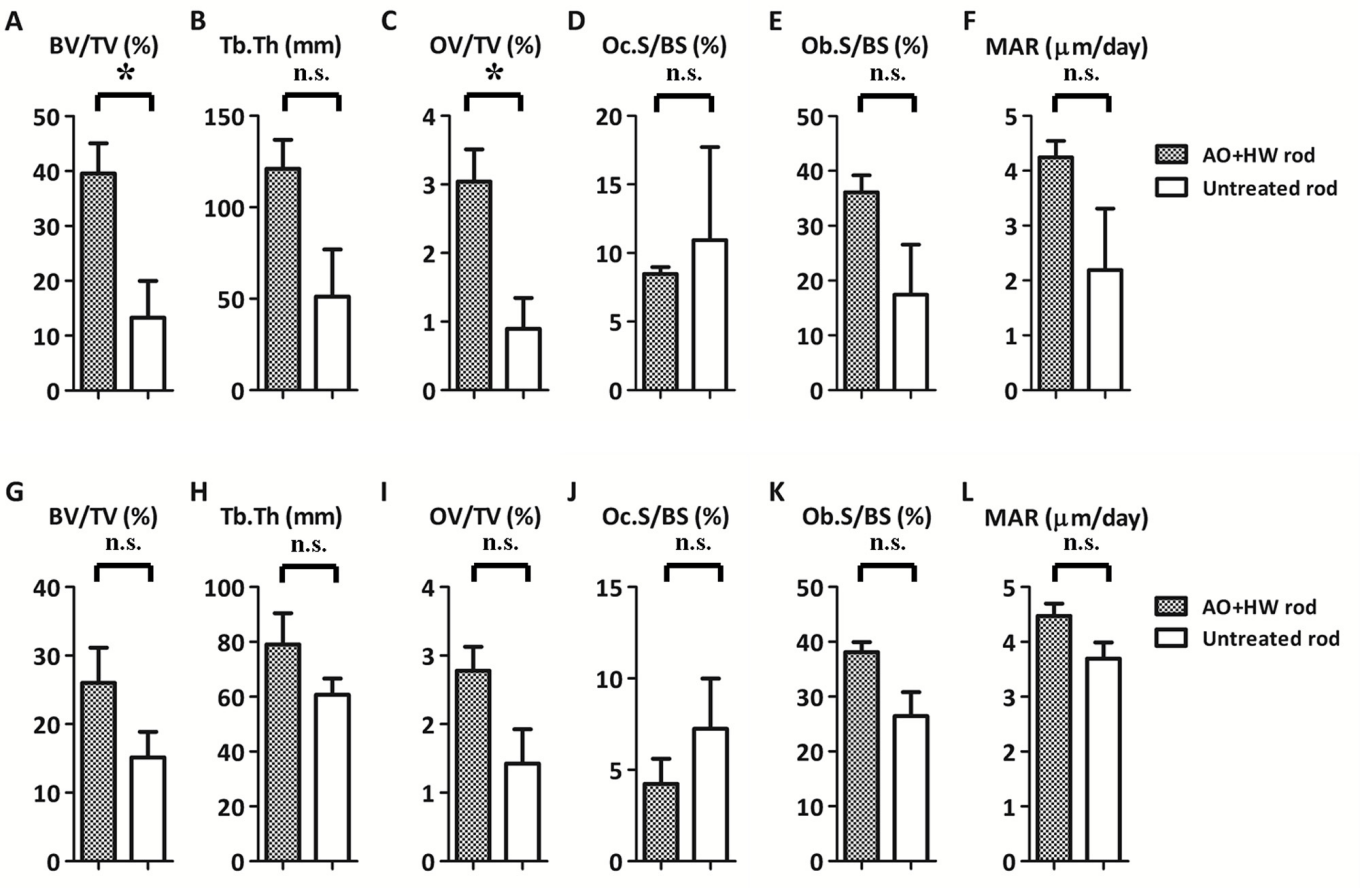
Quantitative histomorphometric analysis of newly-formed bone. Quantitative histomorphometric analysis of newly-formed bone around Ti-Nb-Sn alloy rods 6 weeks after implantation. Parameters from A to F indicate the results of analysing distal areas and parameters from G to L indicate results from proximal areas. BV/TV and OV/TV values in the distal area were significantly higher in AO- plus HW-treated Ti-Nb-Sn alloy rods than in untreated rods (A, C). (*: p < 0.05; n.s.: not significant).

Fig 7 shows representative histological sections for each group. AO- plus HW-treated rods showed more newly-formed bone, especially in the distal femur, as compared with the untreated rods. Fig 8 shows results of quantitative histomorphometric analyses. In the area of the distal femur, the values of BV/TV in AO-plus HW-treated rods (39.56 ± 9.54%) was significantly larger (p < 0.05) than in untreated rods (13.26 ± 11.64%). In the distal area, the OV/TV value in the AO- plus HW-treated rods (3.04 ± 0.81%) was significantly greater (p < 0.05) than in untreated rods (0.90 ± 0.78%). The untreated and treated groups showed no significant differences in the values of Tb. Th, Oc.S/BS, Ob.s/BS and MAR. These results suggested that AO- plus HW-treatment effectively increased new bone formation on the surface of Ti-Nb-Sn rods when distal areas were analysed. There were, however, no significant effects of AO- plus HW-treatment evident in analyses of proximal areas.

## Discussion

In as-anodized samples, crystallization of titanium oxide was not completed, as indicated by a lack of the corresponding diffraction peaks (Fig 3), while XPS analysis indicated existence of both titanium and oxygen on the surface (Table 1, Fig 4). AO treatment induced TiO_2_ formation on the Ti surface and the crystal structure of TiO_2_ varied with electrochemical conditions during AO. TiO_2_ exhibits photocatalicity and superhydrophilicity, and it is used as an environmental photocatalyst for degrading toxic organic pollutants, bacteria and viruses absorbed on its surface under ultraviolet (UV) light illumination. It was reported that UV illumination of a single crystal TiO_2_ generated the shoulder band of the O1s XPS spectrum because of adsorption of hydroxyl groups and water molecules [35]. Cui *et al*. reported that adsorbed hydroxyl groups on the surface of the anodized TiO_2_ with anatase structure were responsible for apatite nucleation and formation in SBF. Subsequent HW treatment after AO could induce this amorphous structure to form an anatase titania [29]. Specific structures of the titania, such as anatase or rutile with high crystallinity, are required for hydroxyapatite nucleation [28] [36]. We propose that HW treatment promoted adsorption of hydroxyl groups and water molecules on anodized TiO_2_, enhancing apatite formation.

Several studies reported that nanosize anatase spheres precipitated on the titania layer after HW-treatment [29] [37]. This effect was described as a dissolution-precipitation mechanism [38] [39]. In this mechanism, initially, the amorphous titania layers began dissolving, resulting in concentrated Ti (IV) in solution close to the samples. Subsequently Ti (IV) started to precipitate on the surface of the substrates because the lower binding energy of the Ti-O bond favours arrangements that crystallize. This phenomenon had previously been proposed to occur with CP-Ti [29] [40] [41]. In our study, titania layers of both CP-Ti and Ti-Nb-Sn alloy were covered by numerous small spheres after HW-treatment. This dissolution–precipitation mechanism also applied to our new β-type Ti-Nb-Sn alloy, as observed with FE-SEM.

It was also proposed that apatite nucleation is induced by abundant titanium hydroxide groups on the surface of titanium [42]. Titanium hydroxide groups on the surface of the titanium alloy influence surface electric charge (zeta-potential) properties [43] [44]. The surface of titanium with abundant titanium hydroxide groups is negatively charged in Hank’s solution and interacts with positively charged Ca^2+^ ions. Therefore, surfaces of titanium substrates become positively charged, leading them to combine with negatively charged nitrate ions in the Hank’s solution. Finally, surface calcium phosphate compounds transform to crystalline apatite. Several studies reported that HW-treatment of a titanium substrate led to formation of abundant titanium hydroxide groups on its surface [29, 41]. As shown in Table 1, the OH- fraction in O 1s XPS did not change after HW treatment in both CP-Ti and Ti-Nb-Sn significantly. The fraction of water molecules in O 1 s XPS significantly increased after HW treatment in both CP-Ti and Ti-Nb-Sn alloy. We believe that both water molecules and hydroxyl groups were responsible for apatite formation. The lower fraction of OH- and water molecules in O 1s XPS for Ti-Nb-Sn alloy, compared with that for CP-Ti, is attributed to the oxide composition in the anodic oxide. This is monolithic TiO_2_ in CP-Ti, while it is Nb_2_O_5_ and SnO_2_/SnO, in addition to TiO_2_, in Ti-Nb-Sn alloy. Nb_2_O_5_ and SnO_2_/SnO do not exhibit hydrophilicity, providing a reasonable explanation for our findings. Here, it should be noted that HW treatment enhanced adsorption of water molecules and hydroxyl groups on the anodized oxide, irrespective of the substrate.

In a previous study, bone-bonding abilities of Ti–6Al–4V and Ti-Nb-Sn alloys were assessed by pull-out strength with rabbit femurs [22]. The failure load of the Ti–Nb–Sn alloy was not significantly different from that of the Ti–6Al–4V alloy. Jinno *et al*. [45] evaluated bone compatibility of Ti–6Al–4V and Co–Cr alloys. They found that the bone–implant interfaces of the Ti–6Al–4V alloy rods had significantly higher shear strength as compared with the Co–Cr alloy rods. In our study, the failure loads of the anodized Ti–Nb–Sn alloy rods after AO- plus HW-treatment were of significantly higher force than those of untreated Ti–Nb–Sn alloy rods. Therefore, we concluded that bone apatite formation on the surface of the Ti-Nb-Sn alloy, induced by AO and HW treatment, improved its biocompatibility with bone.

Our histomorphometric analyses showed, in AO- plus HW-treated groups versus untreated groups, a significant increase in BV/TV and OV/TV values obtained by histological assessment of the distal femur. In analyses of the proximal femur, BV/TV, Tb.Th, OV/TV and Ob. S/BS showed a tendency to increase in the treated group, though the differences did not achieve statistical significance. These results suggested that AO- plus HW-treatment of Ti-Nb-Sn rods induced more formation of new bone. This is consistent with the results of pull-out tests, showing that the failure loads of AO- plus HW-treated Ti-Nb-Sn rods were significantly higher as compared with those of untreated rods. Bone resorption parameters did not significantly differ between AO- plus HW-treated and untreated groups. We propose that the AO- plus HW-treatment stimulated osteoblasts and bone formation around the implanted Ti-Nb-Sn alloy rods, with apatite formation, but did not affect inflammation and osteoclast formation. MAR values, representing the linear production rate of calcified bone matrix, did not differ significantly between the two groups. AO- plus HW-treatment enhanced osteoblast formation around the implanted Ti-Nb-Sn alloy rod but did not affect mineral apposition.

In conclusion, our study showed that AO with acetic acid plus HW treatment improved apatite formation and bone-bonding abilities of the Ti-Nb-Sn alloy. These results increase our understanding of how to improve biocompatibility of orthopaedic prosthesis. For example, AO- plus HW-treated Ti-Nb-Sn is a promising material to improve early bone-bonding.
